# Rapid Renal Alpha-1 Antitrypsin Gene Induction in Experimental and Clinical Acute Kidney Injury

**DOI:** 10.1371/journal.pone.0098380

**Published:** 2014-05-21

**Authors:** Richard A. Zager, Ali C. M. Johnson, Kirsten B. Frostad

**Affiliations:** The Fred Hutchinson Cancer Research Center, and the University of Washington, Seattle, Washington, United States of America; University of Sao Paulo Medical School, Brazil

## Abstract

Alpha-1-antitrypsin (AAT) is a hepatic stress protein with protease inhibitor activity. Recent evidence indicates that ischemic or toxic injury can evoke selective changes within kidney that resemble a hepatic phenotype. Hence, we tested the following: i) Does acute kidney injury (AKI) up-regulate the normally renal silent AAT gene? ii) Does rapid urinary AAT excretion result? And iii) Can AAT's anti-protease/anti-neutrophil elastase (NE) activity protect injured proximal tubule cells? CD-1 mice were subjected to ischemic or nephrotoxic (glycerol, maleate, cisplatin) AKI. Renal functional and biochemical assessments were made 4–72 hrs later. Rapidly following injury, 5–10 fold renal cortical and isolated proximal tubule AAT mRNA and protein increases occurred. These were paralleled by rapid (>100 fold) increases in urinary AAT excretion. AKI also induced marked increases in renal cortical/isolated proximal tubule NE mRNA. However, sharp NE protein levels declines resulted, which strikingly correlated (r, −0.94) with rising AAT protein levels (reflecting NE complexing by AAT/destruction). NE addition to HK-2 cells evoked ∼95% cell death. AAT completely blocked this NE toxicity, as well as Fe induced oxidant HK-2 cell attack. Translational relevance of experimental AAT gene induction was indicated by ∼100–1000 fold urinary AAT increases in 22 AKI patients (matching urine NGAL increases). We conclude: i) AKI rapidly up-regulates the renal cortical/proximal tubule AAT gene; ii) NE gene induction also results; iii) AAT can confer cytoprotection, potentially by blocking/reducing cytotoxic NE accumulation; and iv) marked increases in urinary AAT excretion in AKI patients implies clinical relevance of the AKI- AAT induction pathway.

## Introduction

Acute kidney injury (AKI) up-regulates a variety of stress proteins which can serve as biomarkers of that injury, and potentially impact the course of evolving tissue damage. Well documented examples include neutrophil gelatinase- associated lipocalin (NGAL) [1], [2], kidney injury molecule-1 (KIM-1) [3], [4], L type fatty acid binding protein (L- FABP) [5], [6], and heat shock proteins, e.g., heme oxygenase-1 [7]. In a recent series of studies [8]–[10], we made the surprising observation that, in addition to the above, three stress proteins that are either exclusively, or predominantly, expressed in liver (α-fetoprotein, haptoglobin, hemopexin), are also rapidly induced in mouse proximal tubules in response to ischemic and toxic (maleate, glycerol, and cisplatin) AKI. This was denoted by the following observations: i) AKI increased each of their respective mRNAs; ii) a concomitant increase in RNA polymerase II binding to these gene(s) occurred (implying increased transcription); and iii) marked increases in each of these proteins were documented within renal cortical proximal tubules. Of great interest was that the degrees of increase following experimental AKI were comparable to those observed for NGAL, a classic AKI biomarker gene. This underscored the robust nature of these responses [8]–[10]. To determine whether a clinical correlate of these experimental findings existed, urinary levels of α-fetoprotein and haptoglobin were measured in patients with AKI, and marked increases (again, comparable to those seen for NGAL) were observed [9], [10]. Based on these findings, we coined the term “renal hepatization”, i.e., in which the injured kidney assumes selected features of a hepatic phenotype [8]–[10]. That the normally silent albumin gene was also up-regulated in renal cortex following AKI induction [10] further supported the existence of this “renal hepatization” phenomenon.

A fourth hepatic stress protein is α-1 antitrypsin (AAT) [11], [12]. Thus, with acute liver injury, increased AAT production, with corresponding plasma AAT elevations, result. In addition to its well documented actions as a protease inhibitor (most notably, against neutrophil elastase; NE) AAT has been purported to have diverse cytoprotective and anti-inflammatory effects [13]–[20]. Hence, were AAT to participate in the AKI- “renal hepatization” response, it could potentially alter evolving renal damage. Finally, because AAT is a liver-secreted protein, were AKI to increase renal AAT production, and if urinary secretion were to follow, its urinary levels could serve as a biomarker of acute kidney damage.

Given these considerations, the present study was undertaken to address the following three issues: *First*, does AKI induce the AAT gene within renal cortex/proximal tubule cells? *Second*, does an increase in urinary AAT excretion result, thereby rendering urinary AAT concentrations a potential AKI biomarker? and *third*, can AAT exert direct proximal tubule cytoprotective effects, potentially by acting as a protease/neutrophil elastase inhibitor?

## Results

### Renal cortical AAT mRNA and protein levels

**Figure 1:**
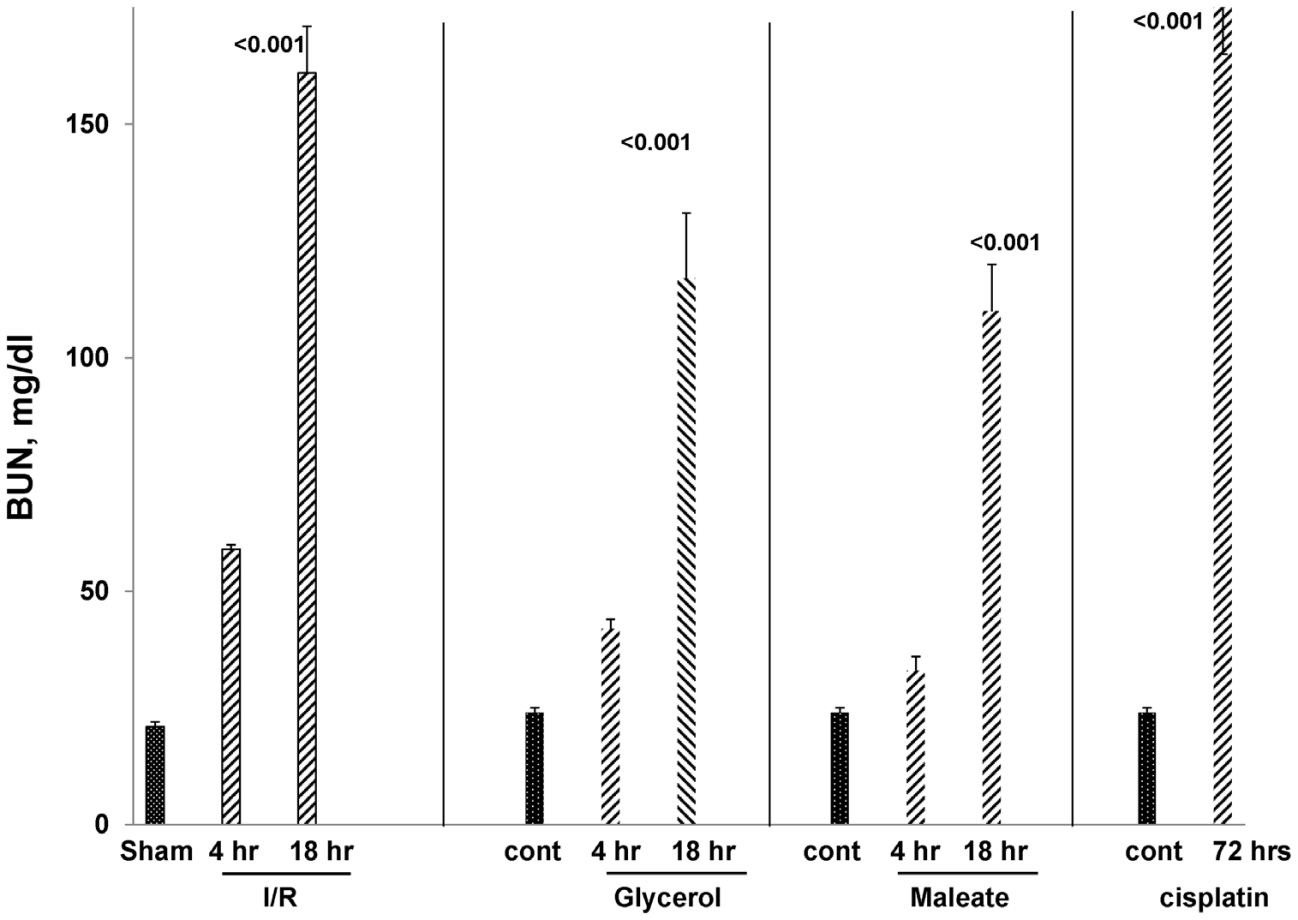
Ischemic and nephrotoxic AKI models induced progressive azotemia. Ischemia/reperfusion (I/R), glycerol induced rhabdomyolysis, and maleate each induced progressive azotemia, as assessed at 4 and 18 hrs post injury induction. Azotemia was assessed only at 72 hrs post cisplatin injection and severe renal failure was observed. Sham  =  sham operated mice, which served as controls for the I/R protocol. Cont  =  control (non operated) mice (n, 5–8 per group; p values vs. sham operated or control mice).

Four AKI models were studied: 30 min of bilateral ischemia/reperfusion (I/R) injury; glycerol- induced rhabdomyolysis; maleate- induced nephrotoxicity; and cisplatin- induced renal failure. Assessments were made at 4 or 18 hrs post induction of I/R, glycerol, or maleate administration. The cisplatin model was studied at 3 days post cisplatin injection because of the slower AKI onset, vs. the other models. Each form of AKI induced severe renal injury, as denoted by progressive azotemia, as depicted in Fig. 1.

#### Renal cortical AAT mRNA levels

**Figure 2:**
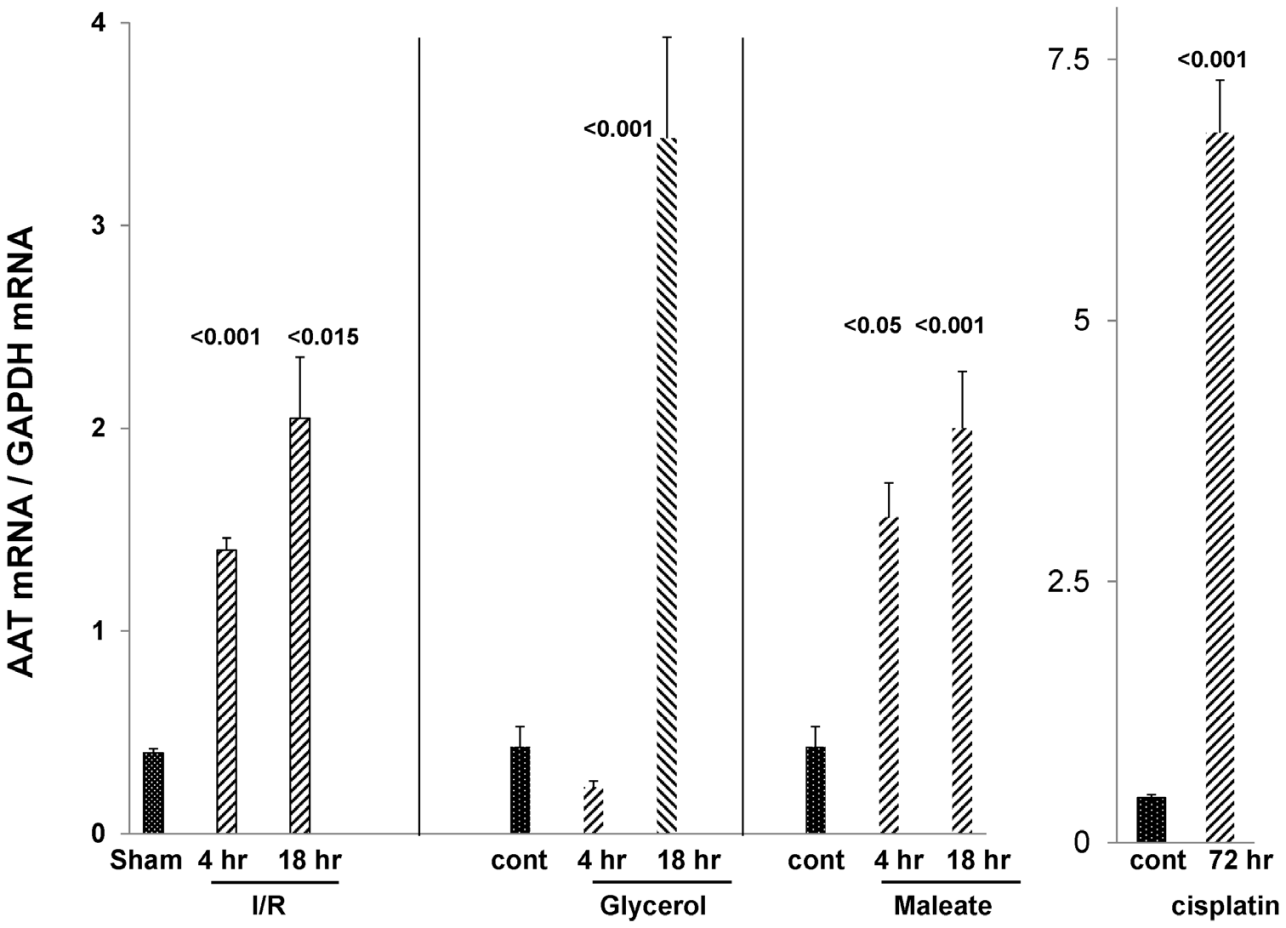
Each of the AKI models induced a marked up-regulation of AAT mRNA levels in renal cortex. I/R approximated tripled AAT mRNA within just 4 hrs of injury induction, with a further increase being observed at the 18 hr time point. The same pattern was observed with maleate. Glycerol evoked an increase in AAT mRNA, but it was slower in onset (seen at 18 but not 4 hrs post glycerol injection). Massive (15 fold) AAT mRNA increases were observed at 3 days post cisplatin injection.

As shown in Fig. 2, each of these injury models induced marked increases in renal cortical AAT mRNA levels. At their height, they rose to approximately 4–15 times basal values. In the case of I/R and maleate induced AKI, the mRNA increases were observed as early as 4 hrs post injury induction, and further increases were documented thereafter (the 18 hr time point).

#### Renal cortical AAT protein levels

**Figure 3:**
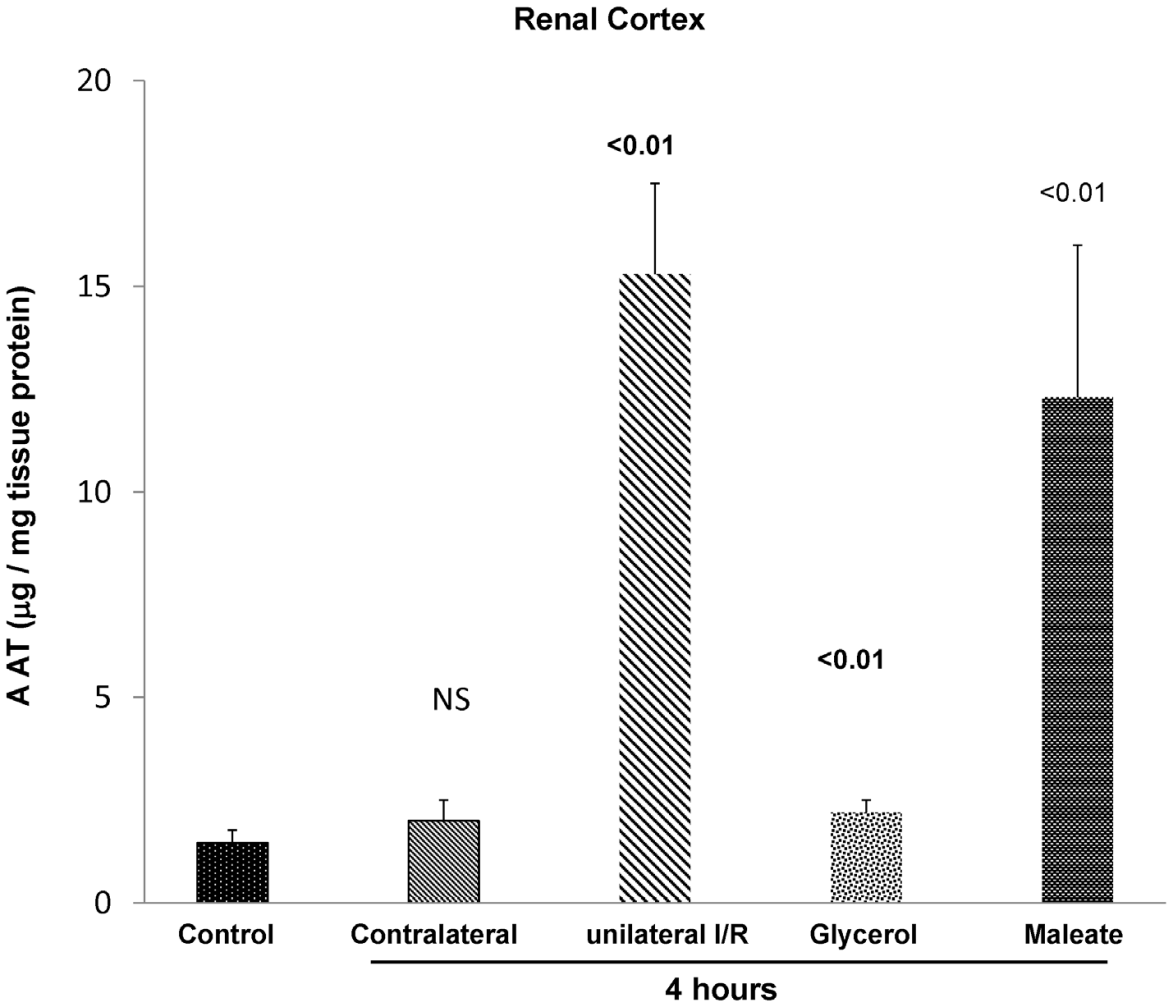
AKI induces a rapid increase in renal cortical AAT protein levels. To assess whether the early (4 hr) AKI- induced AAT mRNA increases were associated with prompt increases in AAT protein levels, the latter were assessed by ELISA. In the case of ischemia/reperfusion (I/R) injury, unilateral ischemia was induced such that the contralateral kidney could serve as a surgical control. Each of the models was associated with significant AAT protein increases, approximately paralleling the relative degrees of AAT mRNA induction, as shown in Fig. 2. Of note is that the contralateral kidney in the unilateral I/R protocol did not manifest an increase in AAT protein levels (discussed in text).

To assess whether the early mRNA elevations were matched by increases in AAT protein levels, the latter were assessed by ELISA at 4 hrs following either unilateral ischemic injury, glycerol injection, or maleate induced AKI. As shown in Fig. 3, by 4 hrs post induction of unilateral ischemic injury, an approximate 15 fold increase in AAT protein levels were observed. Of note was that the contralateral kidney did not manifest an increase in AAT protein levels, compared to normal kidneys. This indicates that the increases seen in the post- ischemic kidney reflected injury, per se, rather than surgical stress, or possible increased AAT uptake from the systemic circulation. Also noted were significant AAT protein increases at 4 hrs post glycerol or maleate injection (∼2 fold and 10 fold, respectively).

**Figure 4:**
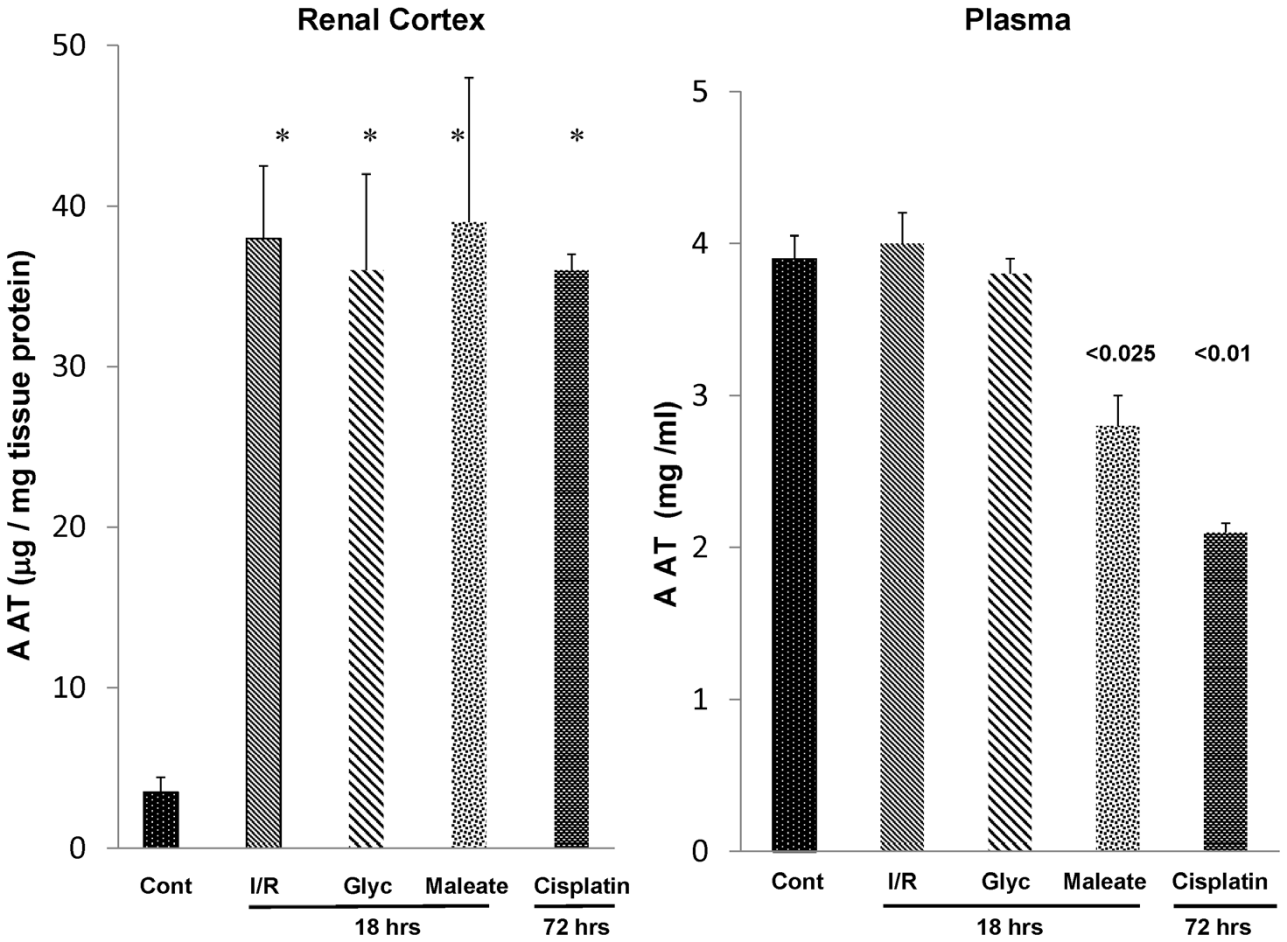
AKI leads to sustained increases in renal cortical AAT protein levels in the absence of any increase in AAT plasma levels. As shown in the left hand panel, approximate 10–20 fold renal cortical AAT protein increases were observed at 18 hrs or 72 hrs post AKI induction. These existed in the absence of any increase in plasma AAT levels (right hand panel). In fact, both the maleate and the cisplatin models were associated with decreased, not increased, plasma AAT concentrations.

Fig. 4 (left panel) presents renal cortical AAT concentrations 18 hrs post bilateral ischemic injury, 18 hrs post glycerol or maleate injection, or 72 hrs post cisplatin administration. Progressive AAT increases over the 4 hr values were observed, reaching levels that were ∼15× normal values. Similarly, by 3 days post cisplatin injection, ∼15 fold renal cortical AAT protein increases were observed.

#### Plasma AAT concentrations and hepatic AAT gene expression

**Figure 5:**
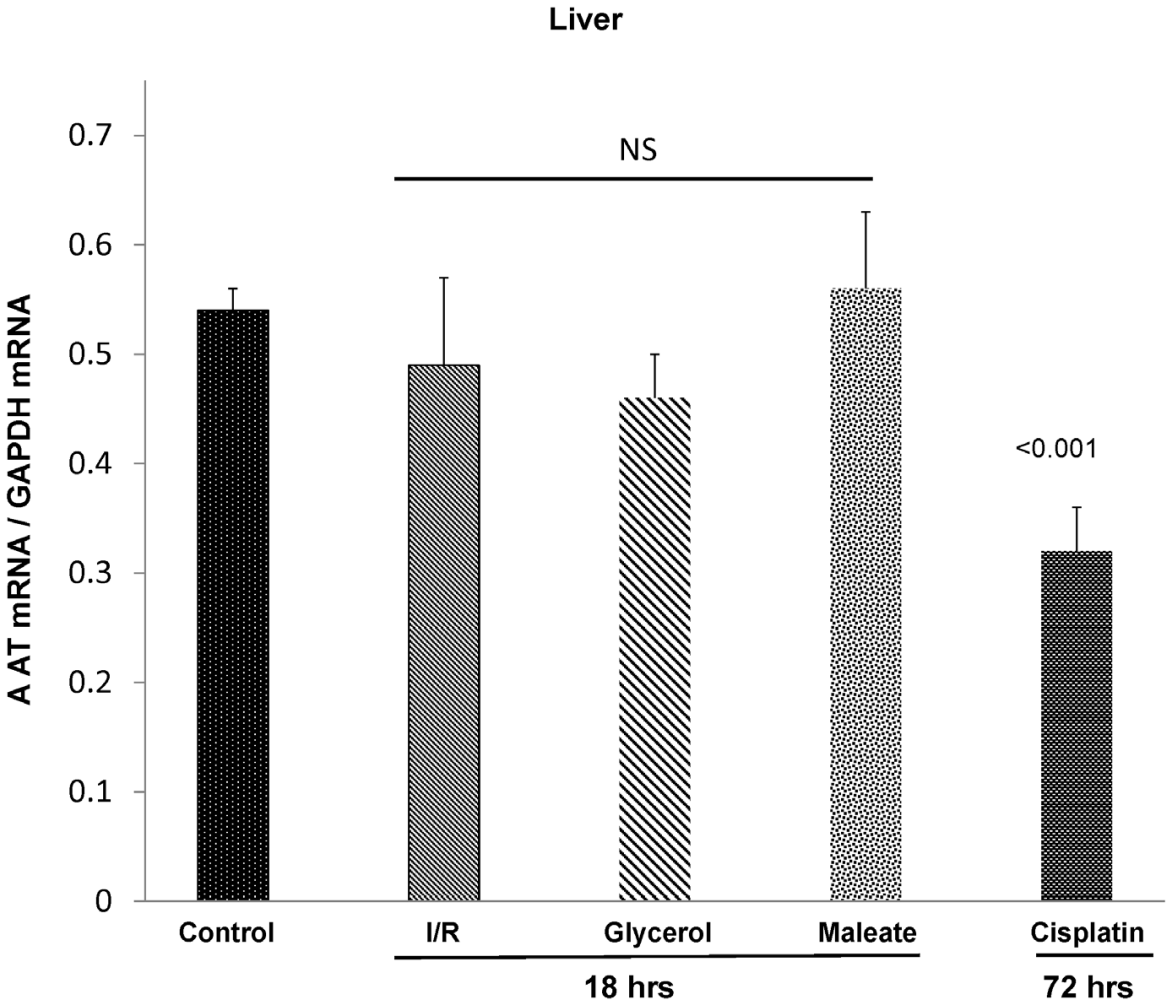
Each of the AKI models failed to increase hepatic AAT mRNA levels. To demonstrate that the renal AAT increases were not associated with increased hepatic AAT production, hepatic AAT mRNA levels were measured. In no case was an increase in hepatic AAT mRNA levels observed. Corresponding with the suppressed plasma AAT levels in the cisplatin model was a significant reduction in its corresponding hepatic AAT mRNA.

To address the possibility that AKI might activate the AAT gene in liver, thereby increasing plasma, and secondarily, renal AAT concentrations, plasma AAT concentrations in each of the above AKI models were assessed. No plasma AAT increases were observed at 18 hrs post I/R or glycerol- induced AKI (Fig. 4, right panel). Paradoxically, plasma AAT concentrations were significantly depressed at 18 hrs post maleate- induced AKI and at 72 hrs post cisplatin injection (Fig. 4, right). Hepatic AAT mRNA levels either remained at, or were below, normal values (Fig. 5). Thus, these findings imply that the renal cortical AAT protein increases almost certainly reflected increased intra-renal AAT production, rather than increased hepatic AAT synthesis with subsequent renal cortical AAT uptake from the systemic circulation. This point is also made by the fact that unilateral ischemic injury only caused renal cortical AAT protein increases in the injured, but not in the uninjured contralateral kidney (Fig. 3).

##### Proximal tubule cell AAT expression

**Figure 6:**
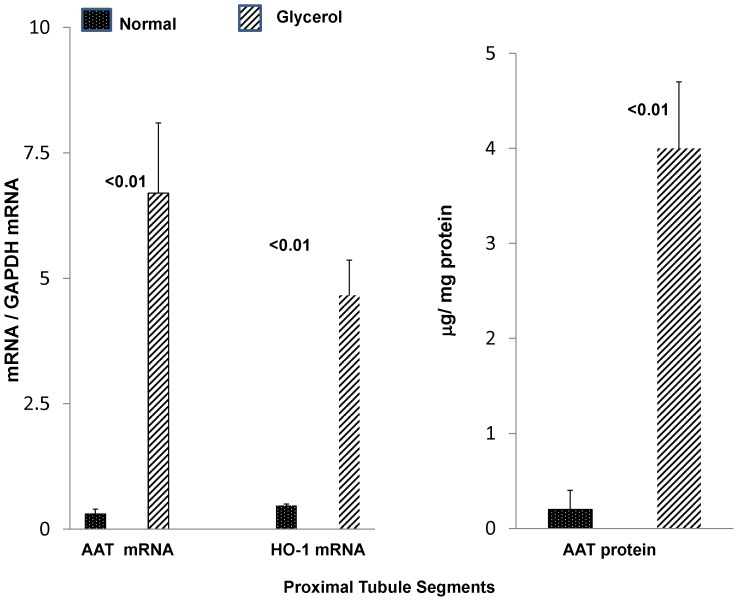
AAT mRNA and protein levels are increased in isolated proximal tubules harvested 18- induced AKI. To demonstrate that the renal cortical AAT changes were reflective of proximal tubule events, proximal tubules were isolated from either normal mice or mice with glycerol induced AKI. AAT mRNA levels were markedly increased in post glycerol exposed tubules, compared to values observed in control tubules (left panel). For comparison, heme oxygenase 1 (HO-1) mRNA, known to be markedly induced in the glycerol model, was also measured and the degree of increase was less than that seen for AAT mRNA. As shown in the right hand panel, a 20 fold increase in AAT protein levels was observed in post glycerol exposed tubules. In sum, these proximal tubule AAT mRNA and protein levels indicate that the renal cortical AAT mRNA and protein increases reflected, at least in part, proximal tubule events.

Given the heterogeneity of cell types within renal cortex, and given that the proximal tubule is the prime cellular target of ischemic and nephrotoxic renal damage, we sought to determine whether the observed renal cortical AAT mRNA and protein increases reflected, at least in part, proximal tubule cell events. To this end, proximal tubule segments were isolated from both normal mice and from mice 18 hrs post glycerol injection, and they were assayed for AAT mRNA and protein levels. As shown in Fig. 6, glycerol induced ∼20 fold increases in AAT mRNA and protein concentrations, compared to values observed in control proximal tubule preparations. The post glycerol mice used for these tubule extractions had severe AKI, as indicated by their corresponding BUN concentrations (141±10 mg/dl) at the time of tubule harvesting (18 hrs).

Because the glycerol AKI model is known to induce a massive increase in HO-1 mRNA, the latter was also measured. As shown in the middle of Fig. 6, the degree of increase of AAT mRNA over control values was ∼ twice as great as that seen for HO-1 mRNA. This underscores the robust nature of the AAT response.

##### Renal cortical and hepatic AAT expression by immunohistochemistry (see Fig. 7)

**Figure 7:**
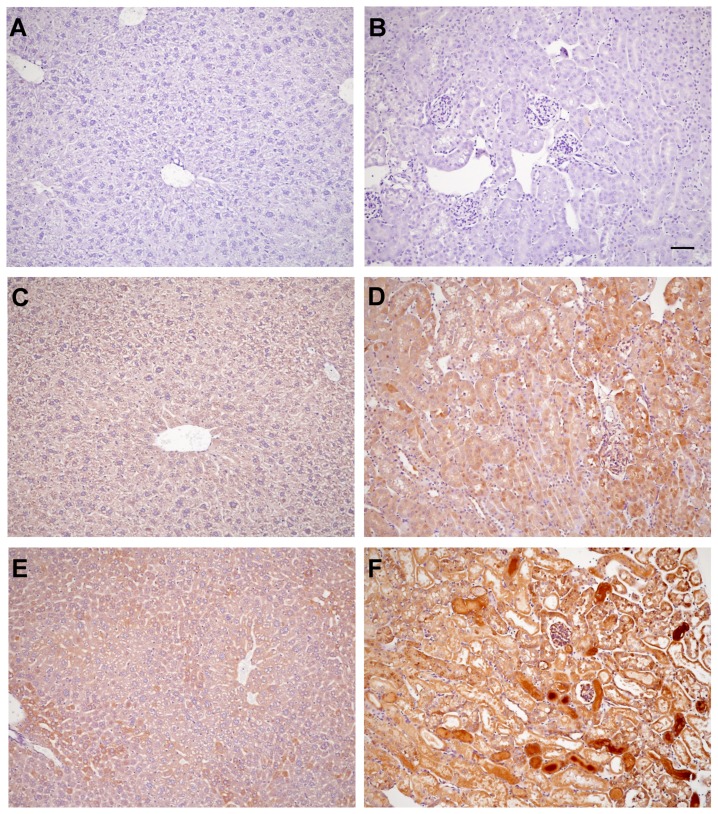
Immunohistochemical localization of AAT in liver and kidney. Panels A and B depict sections from a normal mouse liver and kidney (A, B, respectively) that were stained with an isotype IgG control antibody (1∶250 dilution), serving as negative controls. Panels C and D depict normal liver (C) and normal kidney (D) probed with 1∶250 dilution of anti- mouse AAT. Mild AAT staining was observed, compared to the isotype negative controls. Panel E depicts a liver section from a mouse with glycerol- induced AKI and probed with anti- AAT. No increase in AAT staining is apparent, compared to normal liver (C). Panel F depicts renal cortical AAT staining 18 hrs post induction of glycerol AKI. Marked staining of renal cortical proximal tubule casts and modest cytoplasmic AAT staining within proximal tubules are apparent. The scale bar  = 100 microns.

AAT was detected in both normal liver (panel C) and kidney (panel D), as depicted in Fig. 7 [vs. isotype negative control staining of normal liver (panel A) and normal kidney (panel B)]. AKI did not increase hepatic AAT staining (panel E), consistent with the observation that AKI did not raise hepatic AAT mRNA (Fig. 5). Conversely, AKI increased renal cortical AAT expression, most prominently in renal cortical casts (panel F). A modest increase in proximal tubule cytoplasmic staining was also observed. Thus, these data are consistent with increased tubule AAT production, secretion into tubular lumina, and ultimately, urinary excretion.

##### Urinary AAT (vs NGAL) concentrations following the induction of experimental AKI

**Figure 8:**
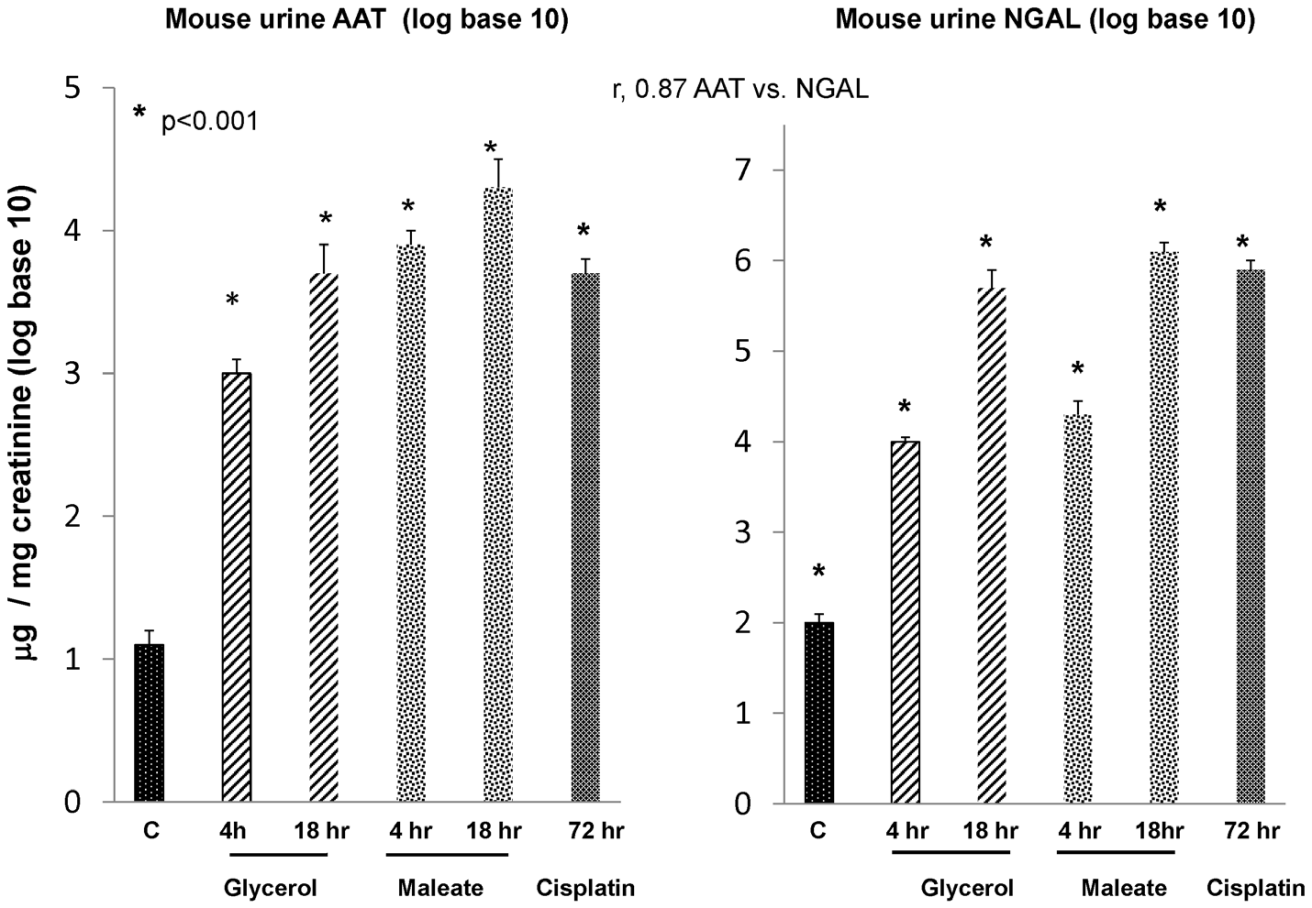
Each of the experimental AKI models induced prompt and massive increases in urinary AAT protein levels, and these increases were comparable to, or exceeded those, observed for urinary NGAL, a classic AKI biomarker protein. All values were factored for urinary creatinine concentrations and are presented as log base 10. Massive urinary AAT protein increases were observed, rising from control values of ∼10 to as high as 30,000 µg/mg creatinine within just 4 hrs of AKI induction (left panel). Furthermore, these values were sustained at each of the tested delayed time points. Of note, these AAT increases were as great, if not greater, than those observed for NGAL (right panel), and a strong correlation (r, 0.87) between AAT vs. NGAL levels was observed.

To assess whether the AKI- induced increases in AAT expression in kidney were associated with increased urinary AAT excretion, AAT levels were measured in urine samples obtained at different time points following induction of the above AKI models. As shown in the left hand panel of Fig. 8, left panel, striking increases in urinary AAT levels (factored by urinary creatinine concentrations) were observed as early as 4 hrs post AKI induction. Because of the massive urinary AAT increases (ranging from normal levels of ∼10 to as high as 30,000 µg/mg creatinine), the data are plotted as log base 10.

As shown in the right hand panel of Fig. 8, the urinary AAT increases were comparable to those observed for NGAL. Furthermore, a strong correlation was observed between AAT/Cr vs. NGAL/Cr levels (r, 0.87). Thus, these findings indicate that: i) AAT is readily secreted from injured tubules (consistent with the immunohistochemistry results); and ii) urinary AAT levels might have early AKI biomarker utility (seemingly comparable in that regard to NGAL).

##### Clinical AKI: urinary AAT vs. urinary NGAL concentrations

**Figure 9:**
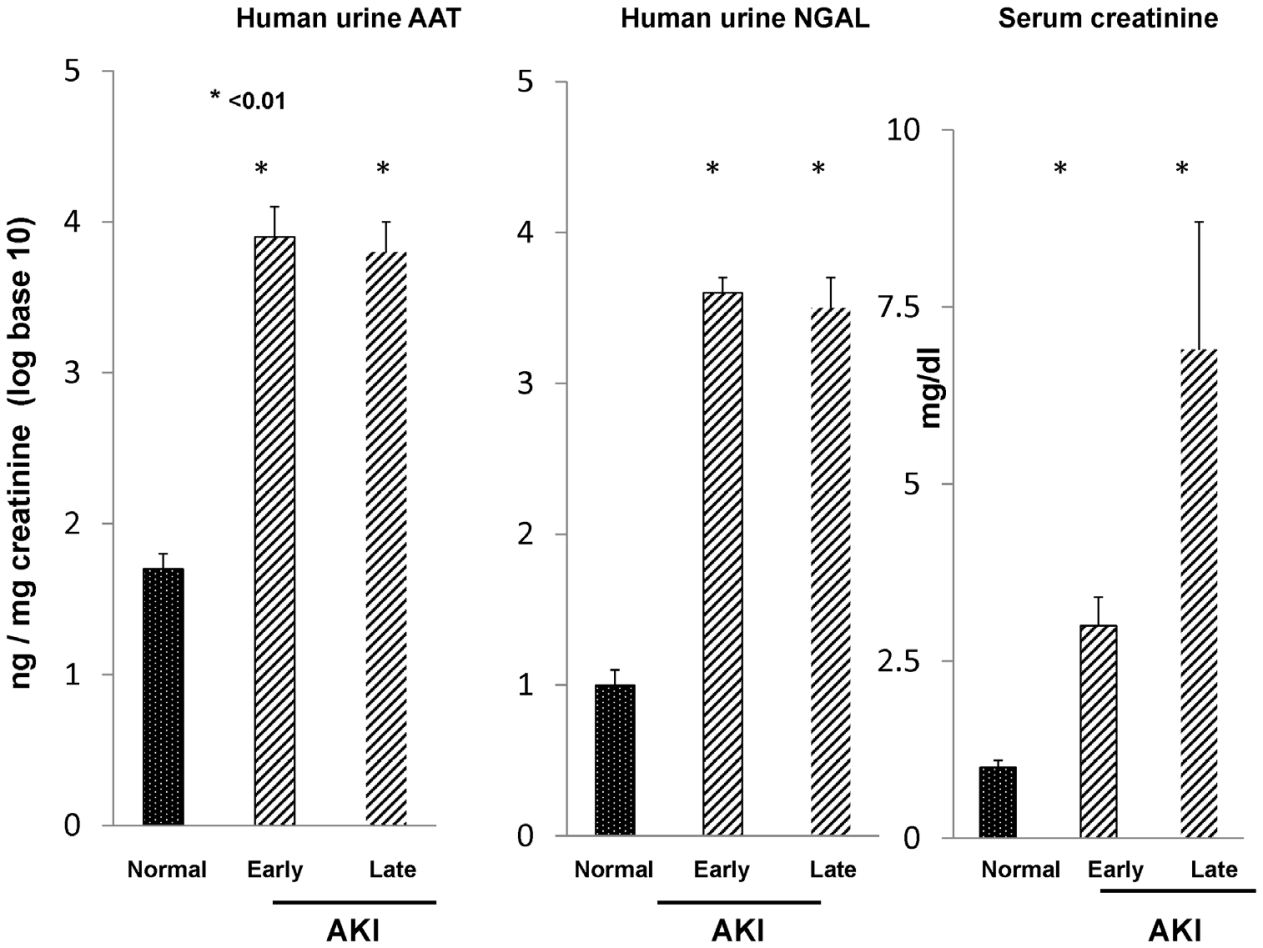
Clinical AKI is associated with massive increases in urinary AAT excretion, and comparable to the increases observed for NGAL. Urine samples were obtained within 48(“early AKI”) or just prior to the start of renal replacement therapy (late AKI). and assayed for urinary AAT and NGAL. Values are given as AAT/creatinine ratios and are presented as log base 10. As shown in the left hand panel, massive and comparable AAT protein increases were observed in the early and late AKI groups, rising from ∼50 to 20,000 ng/mg creatinine. These increases were highly comparable to those observed for urinary NGAL, as shown in the middle panel. Serum creatinines for these groups at the time of urine collection are presented in the right hand panel.

To assess whether a clinical correlate to the above data exists, spot urine samples, obtained from patients with *early* AKI (detected within 2 days of the onset of azotemia [21]), or *late* AKI (samples collected just prior to the onset of renal replacement therapy [9]), were assayed for both AAT and NGAL. As shown in Fig. 9, marked and comparable AAT/Cr and NGAL/Cr increases were observed (as above, the data were factored by urine creatinine and are presented as log base 10). Absolute AAT concentrations ranged from normal values of 56±12 (ng/mg creatinine; 6 normal subjects) to mean values of 21,375 and 22,939 for the early and late AKI groups, respectively.

### Evaluation of renal neutrophil elastase (NE) expression during experimental AKI

Because NE is considered the prime target for AAT mediated protease inhibition [13]–[18], we assessed whether: 1) AKI up-regulates renal cortical NE gene expression (assessed by NE mRNA levels); 2) whether NE is cytotoxic to proximal tubule cells; and 3) whether AAT might exert cytoprotection via its NE inhibitory effect.

#### Renal/proximal tubule NE mRNA levels

**Figure 10:**
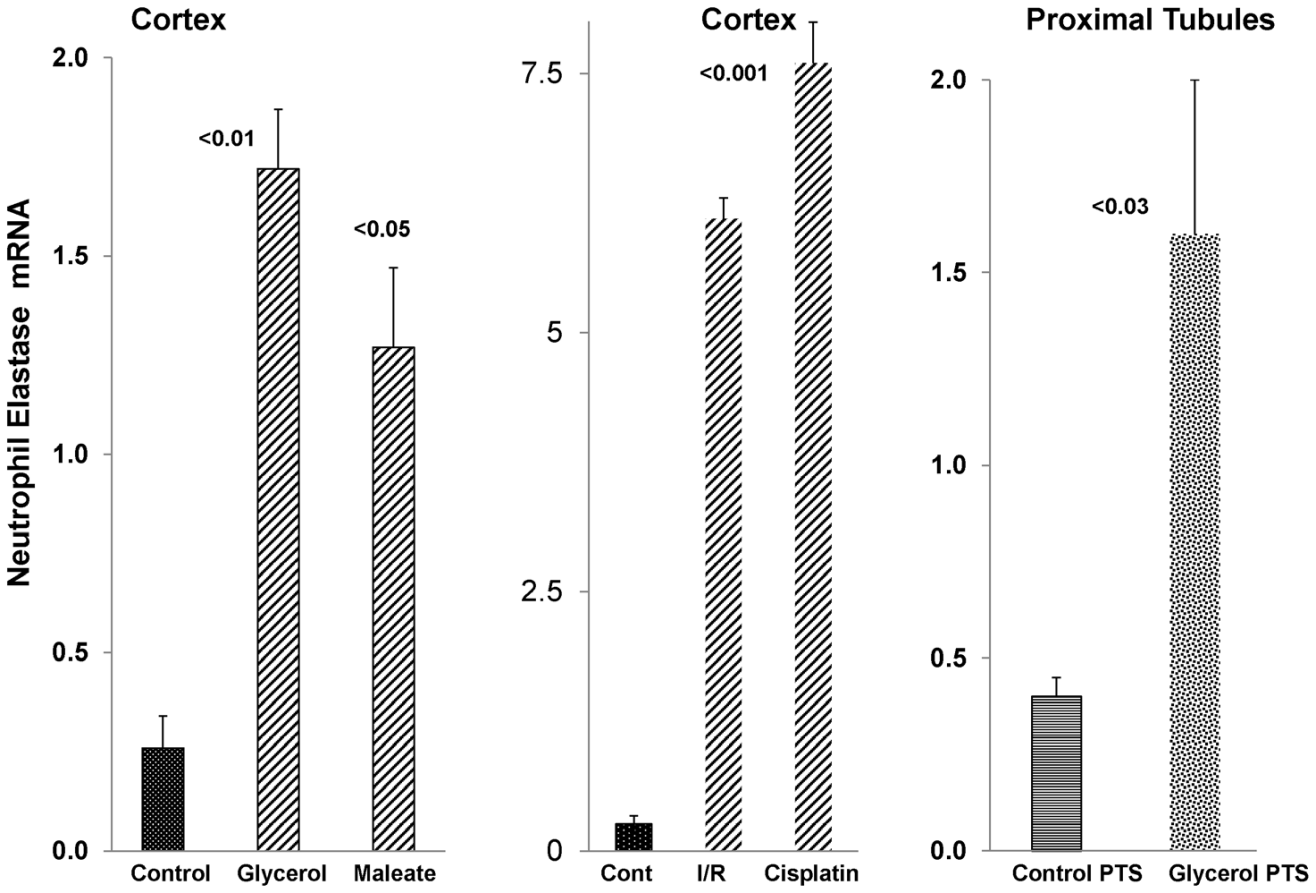
Neutrophil elastase (NE) mRNA levels in renal cortex and in isolated proximal tubules following acute kidney injury. At 18∼3–4 fold increases in NE mRNA levels were observed. In contrast, by 18 hrs post ischemic-reperfusion injury or 72 hrs post cisplatin injection, even more dramatic (∼30 fold) NE mRNA elevations were observed. As shown in the right hand section of the figure, NE mRNA levels in isolated tubules harvested from glycerol treated vs control mice demonstrated an approximate 4 fold increase in the glycerol group. These matched the increases seen in post glycerol AKI whole renal cortex. In sum, AKI caused dramatic increases in renal cortical NE mRNA levels, and these almost certainly stemmed, at least in part, from increases in proximal tubules.

As shown in Fig. 10, each of the experimental AKI models was associated with dramatic increases in NE mRNA. While some of this mRNA increase could have resulted from an influx of neutrophils into the damaged kidney, at least some reflected proximal tubule NE gene induction. This was indicated by 4 fold NE mRNA elevations in isolated proximal tubule segments harvested from kidneys subjected to glycerol induced ARF (Fig. 10, right).

#### Renal NE protein levels

Although an increase in NE mRNA levels would ordinarily be predicted to increase NE protein levels, concomitant AAT protein increases would be expected to covalently bind to NE, and ultimately lead to its degradation [13], [14]. In each of the AKI models, as well as in isolated proximal tubules from glycerol treated mice, striking inverse correlations between AAT and NE protein levels were observed (r values, −0.91 to −0.94), leading to ∼35–50% decreases in NE protein levels.

**Figure 11:**
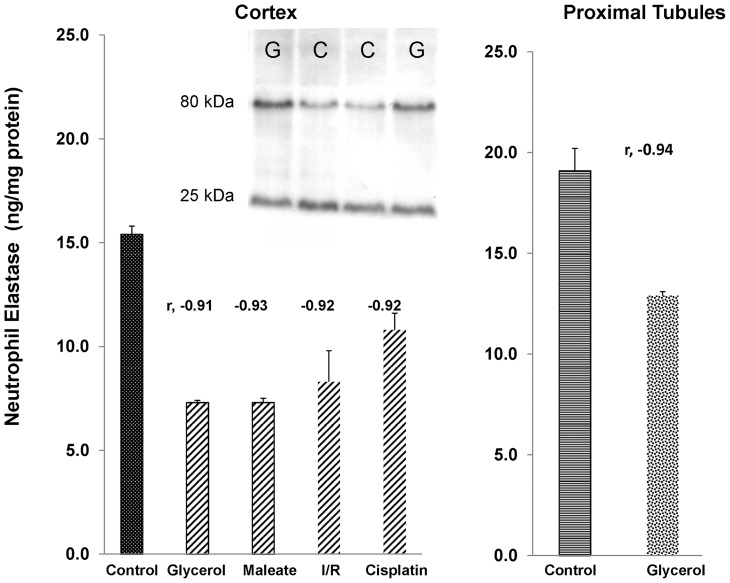
Neutrophil elastase protein levels in renal cortex following different models of experimental AKI. Despite the AKI induced increases in NE mRNA, decreases in NE protein levels were observed. There were striking inverse correlations between NE and AAT protein levels (negative r values shown above each bar). This implies that rising AAT levels led to NE destruction due to covalent binding and subsequent proteolysis [3], [14]. The insert shows the results of Western blotting of control and post glycerol harvested proximal tubule segments. Injury increased the 80 kDa band (bound NE), and modestly decreased (∼35%) the free NE (25 kDa) band. This led to a doubling of the 80 kDa/25 kDa ratio with injury (see text).

Because AAT is known to covalently bind NE and lead to its ultimate proteolytic disruption (13,14), the above proximal tubule segment protein extracts were subjected to Western blotting. A shown in the insert in Fig. 11, increased amounts of a ∼80 kDa band, consistent with NE-AAT covalent binding, was detected with anti-NE antibody. To quantify the relationship between free (25 kDa) vs. bound (80 kDa) NE, as detected by anti-NE antibody, the ratios between the densities of the 80 kDa band were divided by their matched 25 kDa band. The ratios rose from control values of 0.50±0.04 to 1.03±0.16 with glycerol mediated injury (p<0.045). Thus, these results support the concept that with rising AAT protein levels, NE binding occurs, shifting the 25 kDa band to ∼80 kDa (NE, 25 kDa+AAT, ∼55 kDa = 80 kDa).

##### NE induced proximal tubule injury and protection with AAT

**Figure 12:**
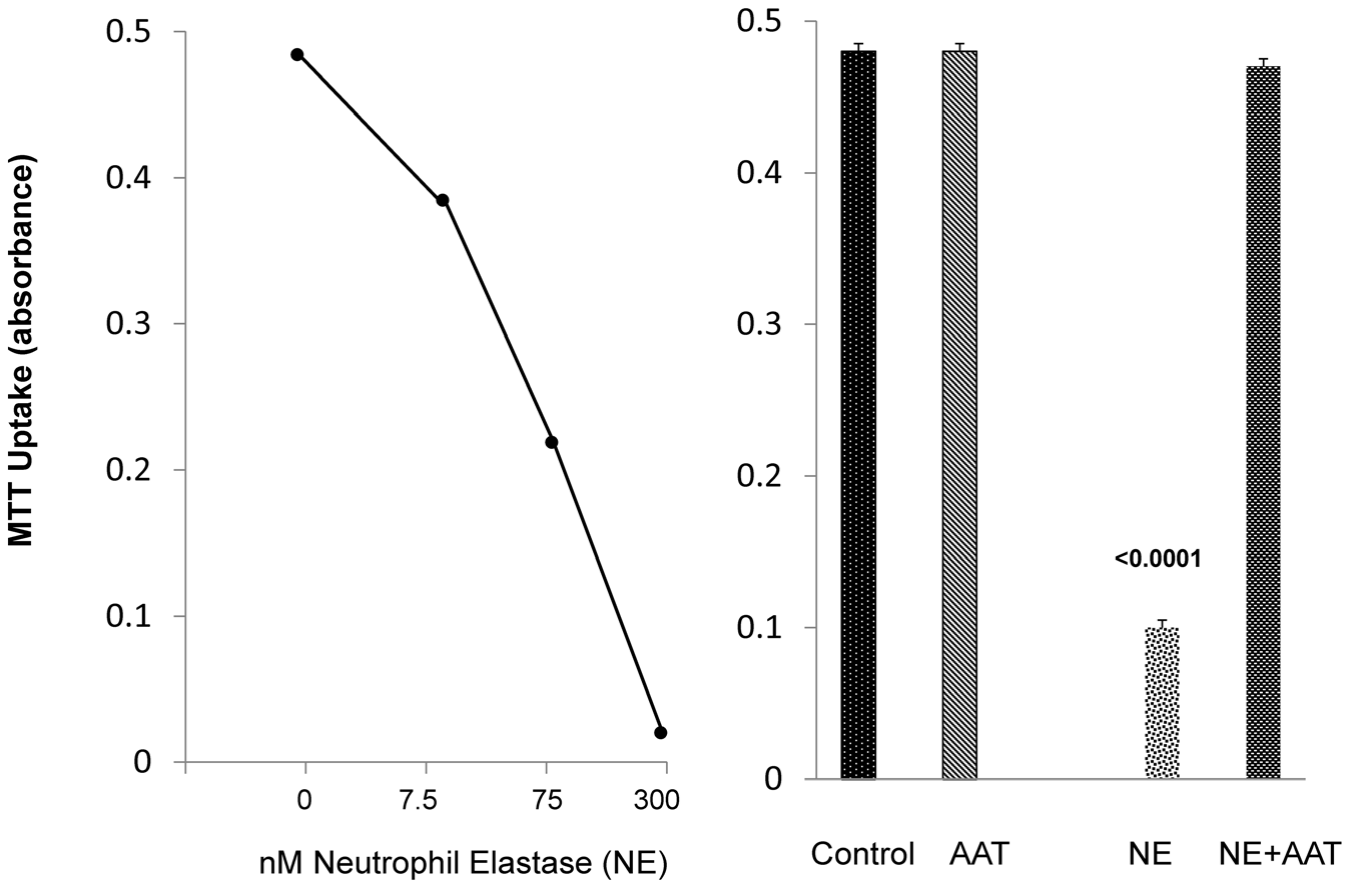
NE mediated cytotoxicity in HK-2 cells, and protection against NE toxicity with AAT. Addition of purified NE induced dose dependent cytotoxicity in HK-2 cells after 18 hr incubations. This toxicity was completely inhibited by concomitant incubation with AAT.

To evaluate whether NE accumulation might exert nephrotoxic/cytotoxic effects, increasing doses of purified NE were added to cultured HK-2 proximal tubule cells and cell viability was assessed 18 hrs later by MTT [3-(4,5-dimethylthiazol-2-yl)-2,5-diphenyltetrazolium bromide] assay. As shown in Fig. 12, a steep dose- response relationship between cell viability and NE concentrations was observed. AAT addition was able to completely block NE's cytotoxic effect (Fig. 12, right).

##### Evaluation of potential protective effects against in vitro proximal tubule injury

**Figure 13:**
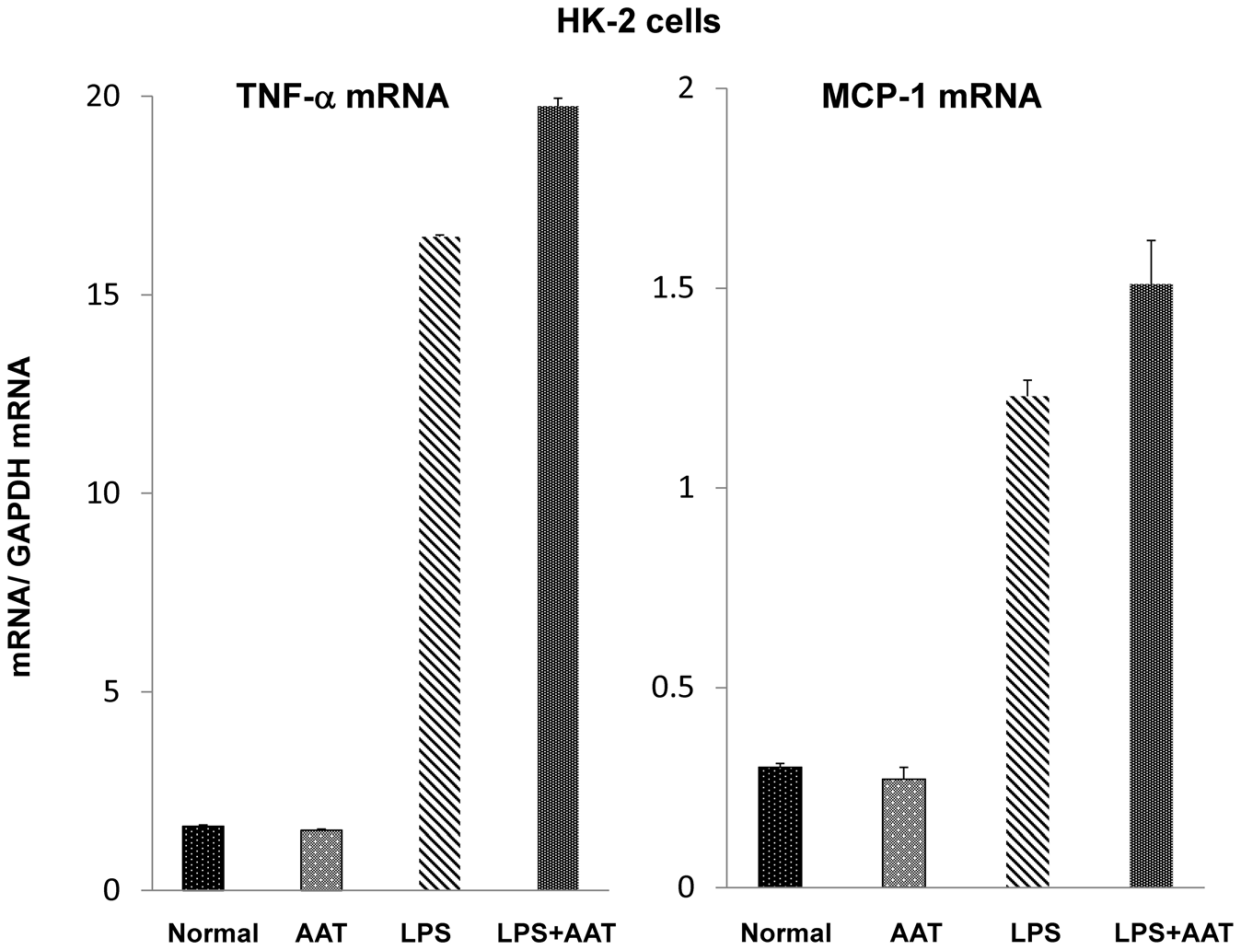
AAT and protease inhibitors confer protection against Fe mediated oxidant HK-2 cell attack. After an overnight incubation with either 1/ml of purified human AAT or a protease inhibitory “cocktail”, HK-2 cells were challenged with Fe mediated oxidative stress. The severity of injury was assessed by MTT assay either 4 or 18 hrs post Fe addition. Both AAT and the protease inhibitors (PI; 0.01% and 0.1%) conferred significant protection against the Fe challenge, as indicated by significant preservation of MTT uptake.

To further evaluate AAT's potential protective actions, HK-2 cells were challenged with oxidative stress, imposed by the addition of ferrous ammonium sulfate in the presence or absence of purified human AAT. To assess whether potential AAT- mediated protection likely reflects its anti-protease activity, the Fe challenge was repeated in the presence or absence of a commercially available protease inhibitor cocktail that does not contain AAT. As shown in Fig. 13, AAT conferred marked protection, as gauged by MTT assay at either 4 hrs or 18 hrs post Fe addition. AAT did not exert an independent effect on the MTT assay. The non AAT containing protease inhibitor (PI) cocktail also conferred dose dependent protection (Fig. 13). This suggests that protease activity is a potential mediator of Fe induced cell injury, and thus, AAT might confer protection via its anti-protease effects.

## Discussion

AAT is an acute phase reactant glycoprotein that is primarily synthesized in hepatocytes and is released into the systemic circulation [11], [12]. Although it may also be produced in small amounts in a variety of extra-hepatic tissues (e.g. neutrophils, lymphocytes, mononuclear phagocytes, enterocytes, pulmonary alveolar cells) [22]–[24], renal tubular cell AAT transcription under basal or stress conditions has not been previously described. AAT's primary biologic activity is as a protease inhibitor [19], [25]. Its best recognized action is directed against NE, although more pleomorphic protease inhibitory activities have also been described [19], [25]. In addition, a variety of non-protease inhibitory effects have been reported, including anti-inflammatory, anti-oxidant, and anti-apoptotic properties [13]–[20]. In sum, these actions have been suggested to evoke a “pro-survival” state. In support of this concept are observations that exogenous AAT administration can protect against endotoxemia [16], pulmonary inflammation [26], [27], graft vs. host disease [18], as well as ischemic renal damage [20]. That hereditary AAT deficiency culminates in chronic pulmonary inflammation/emphysema and hepatocellular injury/cirrhosis underscore AAT's important biologic roles 13,14, 19].

These observations stimulated us to test whether the renal AAT gene might be induced by either ischemic or toxic AKI, and thus, be a component of the “renal hepatization” response. If so, then its purported “pro-survival” activities might have relevance to AKI in at least two important respects: first, if AKI were to acutely induce AAT, it could potentially mitigate evolving renal damage; and second, if injury- induced renal AAT elevations were persistent in nature, it could potentially contribute to so called “ischemic pre-conditioning” or the “cytoresistant” state. In light of these considerations, the present study assessed renal AAT expression during the so called ‘initiation’ and ‘maintenance’ phases of AKI, and tested whether AAT might exert cytoprotective influences directly upon proximal tubule cells.

As shown in Fig. 2, within just 4 hrs of inducing either ischemic or nephrotoxic AKI, a dramatic increase in renal cortical AAT gene transcription was observed, as denoted by ∼3–4 fold AAT mRNA increases. To assess the durability of this response, AAT mRNA levels were also measured in the delayed aftermath of injury induction (18 hrs post I/R, glycerol, maleate AKI; 72 hrs post cisplatin AKI), and further mRNA elevations were observed, reaching values that were ∼10–15× basal levels. To determine whether these mRNA increases resulted in increased translation, AAT protein levels were assessed during the initiation and maintenance AKI phases. Within just 4 hrs of inducing AKI, ∼2–15 fold AAT protein elevations were noted, and as with the mRNA levels, progressive AAT protein increases developed thereafter. Thus, by the 18 hr or 72 hr (cisplatin) time points, AAT levels rose ∼10–20 fold over baseline values. Notably, none of the AKI models increased hepatic AAT mRNA expression. Furthermore, AKI did not raise plasma AAT levels. In fact, in the case of maleate and cisplatin, decreased, not increased, plasma AAT concentrations were observed. Given these hepatic mRNA and plasma AAT results, and the marked renal cortical AAT gene induction (mRNA increases), it is implausible that the AKI- induced renal cortical AAT protein elevations resulted from increased renal AAT uptake from the systemic circulation. Two additional points underscore this point. *First*, the profound AKI- induced reductions in GFR, as denoted by BUN elevations (Fig. 1), would be expected to decrease, not increase, AAT filtration, and hence, renal AAT uptake. *Second*, that unilateral ischemic injury markedly increased renal cortical AAT levels in the post ischemic kidney, but not in the uninjured contralateral kidney, indicates that the AAT increments stemmed from renal synthesis. In sum, the above data indicate that AAT is induced in kidney in response to AKI, and as such, it is a newly documented component of the AKI induced “renal hepatization” response.

Given the heterogeneity of cell types within renal cortex, we sought to determine the origin of the AKI- induced AAT mRNA and protein increases. A priori, one would predict a predominately proximal tubule cell location, given that the proximal tubule is the primary site of ischemic and toxic renal damage. To gain direct support for this assumption, renal cortical proximal tubule segments were isolated from either normal kidneys or from kidneys post induction of glycerol- induced AKI and then tubule AAT mRNA and protein levels were assessed. As shown in Fig. 6, ∼20 fold AAT mRNA and protein increases were observed in glycerol injured tubules, vs. tubules harvested from normal mice. That the percent increases in the injured tubules were approximately twice as great as those observed in whole renal cortex strongly implies that the proximal tubule was the dominant (although likely not the only site) of increased renal cortical AAT production.

In light of the fact that AAT is a hepatic secretory protein, we tested whether AKI- induced proximal tubule AAT production might be reflected by an increase in urinary AAT excretion. That immunohistochemistry demonstrated marked increases in proximal tubule luminal AAT content suggested that this might well be the case. To explore whether such a process might render AAT a potentially useful AKI biomarker, we measured urinary AAT levels following induction of our experimental AKI models. Within just 4 hrs of glycerol and maleate injection, ∼100–800 fold absolute increases in urinary AAT/Cr concentrations were observed (Fig. 7, data presented as log base 10). Indeed, the degree of these 4 hr increases were quantitatively as great, or greater, than those observed for NGAL, and a strong correlation between urinary AAT and NGAL levels was observed (r, 0.87). To test the durability of these changes, urinary AAT and NGAL levels were assessed at more delayed time points (18–72 hrs), and again, marked (>100 fold), and highly comparable AAT and NGAL increases were observed (Fig. 7). To test whether a clinical correlate of these observations exist, urine samples, obtained from patients with early [21] (first diagnosed) or late AKI [9] (i.e., prior to the onset of dialysis) were assayed for AAT, and striking (≥100 fold) elevations were observed. Again, the urinary AAT and NGAL quantitative increases were highly comparable. Thus, these results suggest that our experimental findings have relevance to the clinical arena.

In an effort to ascertain potential pathogenic relevance of AKI- induced AAT up-regulation, we tested the hypothesis that AKI might also activate the renal NE gene. The relevance of this hypothesis is that NE is the dominant AAT target, and thus, if NE gene induction were to occur, and if NE were cytotoxic to proximal tubular epithelium, then an up-regulation of AAT might exert a counterbalancing cytoprotective effect. The accumulated data strongly support this hypothesis. Each form of AKI evoked dramatic NE mRNA increases, implying NE gene induction. However, despite these increases, 35–50% NE protein declines were observed. Furthermore, in each AKI model, the NE protein reductions strongly correlated with the degrees of AAT increases (AAT vs. NE concentrations: r, −0.91 to −0.94). This implies that rising AAT levels led to AAT-NE covalent binding (fig 11, insert) with subsequent NE proteolytic destruction (13,14). Supporting this assumption was that decreases in a ∼25 kDa NE band by Western blotting was associated with increases in an ∼80 KdA band, consistent with Ne-AAT binding. The potential pathophysiologic significance of these NE reductions is indicated by our observations that NE is highly cytotoxic to proximal tubule (HK-2) cells, and that this toxicity can be completely blocked by AAT addition.

To test whether AAT's protective action could be expressed against a more general form of cell injury, we challenged HK-2 cells with Fe mediated oxidative stress in the presence or absence of AAT. At both 4 and 18 hrs post Fe addition, AAT markedly decreased the extent of Fe mediated cell death. To test whether this protection likely stemmed from AAT's anti-protease activity, HK-2 cells were again challenged with Fe, but this time in the presence or absence of a protease inhibitor “cocktail” that does not contain AAT. Once again, dramatic protection against Fe cytotoxicity was observed. Thus, these findings imply a critical role played by proteases in the evolution of tubule cell death. Seminal studies from Kaushal and Shah [28] underscore this point. These investigators noted that the addition of meprin, a renal metalloprotease, induced cytotoxicity in MDCK cells [28]. Hence, our current findings that AAT can protect against in vitro cell injury, and that this action may stem, at least in part, by its anti-protease/anti NE activity, are certainly consistent with Kaushal's and Shah's conclusions: i.e., that protease activation can play a critical role in the evolution of AKI mediated tubular cell death.

In conclusion, the present study presents the novel observation that diverse forms of nephrotoxic and ischemic AKI induce rapid and profound AAT gene induction within proximal tubules, culminating in up to 20 fold increases in renal cortical AAT protein levels. A correlate of this process is rapid AAT tubule secretion, leading to marked (∼100–1000 fold) increases in urinary AAT excretion. The pathophysiologic relevance of renal tubular AAT gene induction is indicated by the following: i) renal cortical levels of NE decline during AKI, despite increased NE gene transcription (as expected from rising AAT levels); ii) NE is directly cytotoxic to proximal tubule cells; iii) AAT completely blocks NE cytotoxicity; and iv) AAT can also mitigate Fe mediated oxidant tubular cell attack, likely due to its anti-protease activity. Finally, that AAT urinary excretion rises dramatically in patients with AKI, paralleling NGAL excretion, implies translational relevance of the above experimental observations. Indeed, AAT urinary assay could potentially signal AKI, and thus, have biomarker utility.

## Methods

### Animal experiments

Male CD-1 mice (30–40 grams; Charles River Laboratories, Wilmington, MA) were used for all in vivo AKI protocols. The animal experiments used in this study were specifically approved by the Fred Hutchinson Cancer Research Center's Institutional Animal Care and Utilization Committee (IACUC). The methods for inducing AKI were as follows:

#### Bilateral ischemia/reperfusion induced AKI

Mice were deeply anesthetized with pentobarbital (40–50 mg/Kg) and then both renal pedicles were identified through a midline abdominal incision. Bilateral ischemic injury was induced by cross clamping the renal pedicles for 30 min with atraumatic vascular clamps. Body temperature was maintained at 36–37°C by use of an external heating lamp. Following clamp removal, uniform reperfusion was confirmed by the loss of renal cyanosis and then the abdominal incisions were closed in two layers with silk sutures. Sham operated mice served as controls. Eighteen hours post surgery, the mice were re-anesthetized, the abdominal cavities were opened, a urine sample was expressed from the urinary bladder, a blood sample was obtained from the inferior vena cava, and then the kidneys were resected. The kidneys from each animal were iced, the renal cortices were resected, and total mRNA (RNeasy; Qiagen) and total protein extracts were prepared. The RNA samples were assayed for AAT and GAPDH mRNAs by competitive RT-PCR, using the primers presented in Table 1. The results were expressed as AAT/GAPDH ratios. The renal cortical protein extracts and urine samples were assayed for mouse AAT by ELISA (Innovative Research, Inc., Novi, MI; Catalog# IRFPN1339). Cortical AAT samples were expressed as µg/mg of extracted protein. The urine AAT values were factored by the urine creatinine concentrations, and the results were expressed as log base 10. For comparison, the urinary AAT levels were compared to those observed for mouse NGAL, as determined by ELISA (R and D Systems, Minneapolis, MN: DY1857). An n of 5–6 samples were obtained from both sham operated and post ischemic AKI mice. The plasma samples were used to measure BUN and AAT protein concentrations (ELISA).

**Table 1:** Primers used to measure mouse AAT and GAPDH mRNAs.

mRNA	Primer Sequences	Product Size
**Mouse AAT**	5′-TTC CAA CAC CTC CTC CAA AC -3′ 5′-CAC CGC CTC AGC TAT CTT TC-3′	234 bp
**Mouse Neutrophil Elastase**	5′-ACT GTG TGA ACG GCC TAA AT -3′ 5′-CTG GGT GAT GGT CTG TTT GT -3′	292 bp
**Mouse GAPDH**	5′-CTG CCA TTT GCA GTG GCA AAG TGG-3′ 5′-TTG TCA TGG ATG ACC TTG GCC AGG-3′	437 bp

Mouse AAT and NE mRNAs were measured by RT-PCR and factored by simultaneously obtained GAPDH product.

#### Unilateral ischemia – reperfusion injury protocol

To assess AAT changes at an earlier time point, the above protocol was repeated in 5 mice with the following changes: 1) left unilateral ischemia was performed, leaving the contralateral kidney intact; and 2) the animals were sacrificed 4 hrs post ischemia induction. The post ischemic renal cortical AAT protein values were contrasted to those found in the uninjured contralateral kidneys.

#### Glycerol induced AKI

Mice were briefly anesthetized with isoflurane, and then 10 ml/Kg of 50% glycerol was administered IM in two equally divided lower limb injections. Either 4 hrs (n, 6) or 18 hrs later (n, 6), the mice were deeply anesthetized with pentobarbital, the abdominal cavities were opened, and then blood, urine, renal cortical protein, and renal cortical RNA samples were obtained and assayed as denoted above. An equal number of normal mice provided control samples for the analyses noted above.

#### Maleate induced AKI

Ten mice were injected intraperitoneally with Na maleate 800 mg/Kg (in ∼0.7 ml saline, pH adjusted to 7.4) or with the same volume of saline vehicle. Either 4 or 18 hrs later, the animals were anesthetized, and blood, urine, and renal cortical samples were harvested for analyses, as noted above. The results were compared with non maleate injected control mice (n, of 5–6 at each time point from maleate and controls).

#### Cisplatin induced AKI

Five mice were injected with 30 mg/Kg cisplatin (in 1 ml of sterile saline). Five saline injected mice served as controls. Three days post injection, the mice were anesthetized with pentobarbital, and plasma, renal cortical, and urine samples were collected. Plasma samples were assayed for BUN and AAT protein levels; renal cortices were assayed for AAT protein and mRNA; and urine samples were assayed for AAT and creatinine levels.

#### Hepatic AAT mRNA levels

To assess the impact of the experimental AKI protocols of hepatic AAT gene expression, pieces of liver were resected from 4 mice subjected to each of the AKI protocols (18 hrs post I/R, maleate, glycerol; 72 hrs post cisplatin) and they were assayed for AAT mRNA. Hepatic samples from 5 control mice provided control AAT mRNA values.

##### Isolated proximal tubule assessments

Renal cortical proximal tubules were prepared from 5 normal mice and 5 mice that had been injected 18 hrs earlier with glycerol, as noted above. In brief, the kidneys were resected, the cortices dissected and diced with a razor blade, and then the tissues were incubated with collagenase [29]. Proximal tubules were isolated via centrifugation through 31% Percoll [29]. Following isolation, protein and RNA were extracted and assayed for AAT (ELISA) and AAT mRNA as done for whole renal cortex. For comparison, AAT mRNA values were contrasted to those for HO-1 mRNA, as determined by RT-PCR [7].

##### Immunohistochemistry

AAT was probed in normal liver, normal kidney, and in liver and kidney from a mouse 18 hrs post glycerol– induced AKI. Two micron paraffin embedded sections were cut and probed with rabbit anti- mouse AAT at a 1∶250 dilution (Biorbyt; Cambridge, Cambridgeshire, United Kingdom; # orb10017). Anti AAT was visualized with anti- rabbit, HRP labelled, IgG (Leica Biosystems; #PV6112, Newcastle Upon Tyne, UK) as previously described [9]. Isotype negative rabbit IgG served as negative liver and kidney controls.

### Impact of AKI on renal expression of neutrophil elastase (NE)

Renal cortical mRNA and protein samples, obtained at 18 hrs post ischemia/reperfusion, glycerol, or maleate induced injury, or 72 hrs post cisplatin injection, were probed for NE mRNA using the primers shown in  1. Protein extracts were assayed for NE using a commercially available ELISA (M7718; TSZ Scientific; Waltham MA). The results were compared to those in control kidney samples. Isolated tubule mRNA and protein samples obtained from control and 18 hr post glycerol mice were also probe for NE protein and mRNa levels.

#### NE Western blotting

Isolated proximal tubule protein samples, harvested from 4 control mice and 4 mice 24 hr post glycerol treatment, were subjected to Western blotting, as previously described in detail [30]. The samples were probed for NE with an anti-NE rabbit antibody from Abcam (Cambridge MS; cat # Ab68672). Antibody detection was performed with horesradish peroxidates linked anti-rabbit IgG (From GE HealthCare, NJ; cat. #NA934)., as previously described [30].

### HK-2 cell experiments

#### NE cytotoxicity

HK-2 cells, a human proximal tubule cell line, were used for these experiments.

The cell line was created as previously described [31]. The cells were originally obtained from a piece of discarded human kidney during the process of kidney transplantation. Informed written patient consent was obtained in accordance with the Fred Hutchinson Institutional Review Board (IRB) for Human Studies which approved this tissue acquisition. HK-2 cells were grown in T75 Costar flasks with keratinocyte serum free medium (KSFM) with added glutamine, pituitary extract, and penicillin/streptomycin as previously described [31]. After reaching near confluence, they were recovered by trypsinization and scraping and re-plated in T48 well Costar plates. After allowing ∼6 hours for cell reattachment, they were challenged with increasing doses of purified NE (0, 7.5, 75, 300 nmoles/ml; NE; Athens Research, Athens, GA EH2009-01). Eighteen hrs later, viable cell numbers were assessed by cellular uptake of the tetrazolium dye MTT [31]. Values were presented as % MTT uptake, compared to that observed in cells incubated under normal incubation conditions (n, 6 determinations each).

#### Protective effects of AAT against NE toxicity

Using the above described protocol, HK-2 cells were challenged overnight with 100 nmoles/ml of NE in the presence or absence of 1 mg/ml of purifed human AAT (Baxter Laboratories, Deerfield IL; “alpha” −1 proteinase inhibitor; from Glassia; NDC 0944-2884-01). The following morning, the extent of cell injury was determined by MTT assay.

#### AAT effects against Fe mediated oxidant cell attack

Given the importance of catalytic Fe in diverse forms of AKI, the impact of AAT on Fe mediated injury was assessed. HK-2 cells were incubated with a pro-oxidant Fe challenge (5 µM ferrous ammonium sulfate, complexed to equimolar hydroxyquinoline; a lipophilic Fe chelator, permitting intracellular Fe access; [32]) in the presence of absence of 1 mg/ml AAT. After completing either 4 hr or 18 hr incubations, the extent of cell injury was assessed by MTT assay, as above.

#### Impact of non AAT mediated protease inhbition on HK-2 cell injury

To assess whether non AAT mediated protease inhibition would also confer protection, the above experiment was repeated with or without the addition of a commercially available protease inhibitor ‘cocktail’ that does not contain AAT (“Complete” #11-697-498-011; Roche Applied Science; Mannheim Germany; final concentrations of 1∶10 and 1∶100 dilutions). Fe mediated cell injury was assessed 4 hrs post Fe addition, as noted above.

### Clinical AKI samples

#### Early AKI

Spot urine samples were obtained from 14 critically ill patients who were hospitalized at Vanderbilt University Hospital ICU, Nashville, TN. They were enrolled in the IRB- approved VALID study [21] and had a diagnosis of early AKI, as denoted by a 33% increase in their serum creatinine concentrations from baseline values. The specifics of this patient population have been previously described [21].

#### Late AKI

Spot urine samples were obtained from 8 patients enrolled in an IRB approved study at Washington University, St Louis, MO who had a diagnosis of late AKI, as defined by progressive azotemia with the requirement for renal replacement therapy. The urine samples were obtained within 12 hrs prior to the onset of dialytic therapy. Specifics of this patient population have also been previously described [9].

#### Control urine samples

Spot urine samples, obtained from 6 normal subjects, were used to establish normal values.

#### Testing

All of the above urine samples were assayed for human AAT and human NGAL using commercially available ELISAs (AAT: R&D Systems, Minneapolis, MN; Catalog # DY1268; NGAL, R&D Systems, Minneapolis, MN; # DY1757). Values were factored by urine creatinine and the ratios were expressed as log base 10.

#### Calculations and Statistics

All values are presented as means ± 1SEM. AAT mRNA levels were quantified by competitive RT-PCR using the primers given in Table 1. The results were expressed as a ratio to simultaneously determined GAPDH cDNA. Statistical comparisons were made by unpaired Student's t test. Significance was judged by a p value of <0.05.
